# Supplementary material for: Identification of photoperiod-induced specific miRNAs in the adrenal glands of Sunite sheep (Ovis aries)
Source: Front Vet Sci. 2022 Jul 22;9:888207. doi: 10.3389/fvets.2022.888207 (PMC9354845; doi:10.3389/fvets.2022.888207)
Supplement: Supplementary file 1 [file Data_Sheet_1.ZIP › Supplementary file/miRDeep2 file.zip!/miRDeep2 file/Novel_Structure/SP42A2/Novel_89.pdf]

Provisional ID	:	NC_019471.2_28095
Score total	:	2.4
Score for star read(s)	:	-1.3
Score for read counts	:	0
Score for mfe	:	2.1
Score for randfold	:	1.6
Score for cons. seed	:	
Total read count	:	102
Mature read count	:	34
Loop read count	:	0
Star read count	:	68

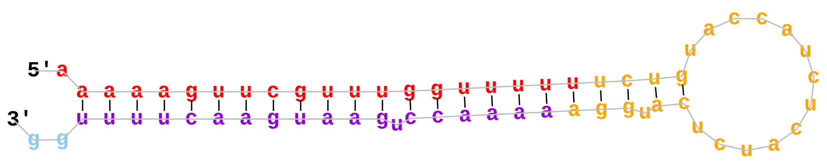

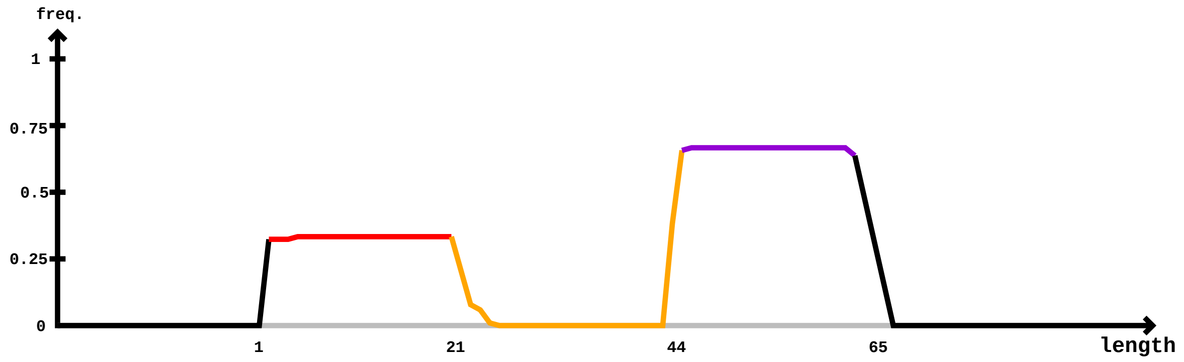


```
                 Mature                                   Star
5'- acuucuguauuggguuggcuaaaaaguucguuugguuuuuucuguaccaucucaucucauggaaaaaaccugaaugaacuuuuggccaacccagcagaaauuaucaaggagga   -3'    obs
    acuucuguauuggguuggcuaaaaaguucguuugguuuuuucuguaccaucucaucucauggaaaaaaccugaaugaacuuuuggccaacccagcagaaauuaucaaggagga          exp
    ..((((((..((((((((((((((.(((((((((((((((((((.............)).))))))))).))))))))))))))))))))))))))))..............    reads    mm    sample
    ....................aaaaaguucguuuggGuuuu........................................................................    23       1     seq
    ....................aaaaaguucguuugguuuuu........................................................................    2        0     seq
    ....................aaaaaguucguuugguuuuuu.......................................................................    1        0     seq
    ....................aaaaaguucguuugguuuuuuc......................................................................    2        0     seq
    ....................aaaaaguucguuuggGuuuuucu.....................................................................    5        1     seq
    .......................aaguucguuuggGuuuuucug....................................................................    1        1     seq
    ..............................................................Uaaaaccugaaugaacuuu...............................    1        1     seq
    ..............................................................aaaaaccugaaugaacuuuu..............................    6        0     seq
    ..............................................................aaaCaccugaaugaacuuuu..............................    1        1     seq
    ..............................................................aaaaaccAgaaugaacuuuu..............................    13       1     seq
    ..............................................................aaaaaccugaaugaacuuuuC.............................    1        1     seq
    ..............................................................aaaaaccCgaaugaacuuuug.............................    14       1     seq
    ..............................................................aaaaaUcugaaugaacuuuug.............................    1        1     seq
    ..............................................................aaaaaccugaaugaacuuuuUgc...........................    2        1     seq
    ...............................................................aaaaccAgaaugaacuuu...............................    2        1     seq
    ...............................................................aaaaUcugaaugaacuuuu..............................    23       1     seq
    ...............................................................aaaaccugaaugaacCuuu..............................    1        1     seq
    ...............................................................aaaaccugaaugaacuuuuC.............................    1        1     seq
    ...............................................................aaaaUcugaaugaacuuuug.............................    1        1     seq
    ................................................................aaaUcugaaugaacuuuuggc...........................    1        1     seq
```
